# Supplementary material for: Clinically advanced NLRP3 inhibitor modulates microglial transcriptome and alleviates α-synuclein-induced progression of parkinsonism
Source: J Neuroinflammation. 2026 Jan 31;23:76. doi: 10.1186/s12974-026-03716-3 (PMC12947520; doi:10.1186/s12974-026-03716-3)
Supplement: Supplementary file 1 — Supplementary Material 1: Fig S1 to S3. [file 12974_2026_3716_MOESM1_ESM.pdf]

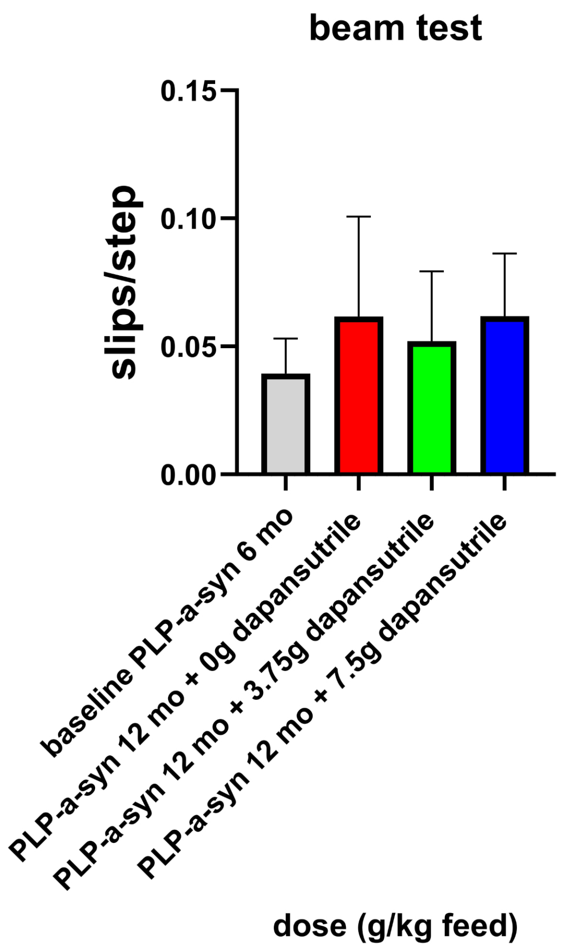

Fig. S1. Challenging beam test performance of PLP-a-syn mice at baseline (6 months of age) and at 12 months of age either untreated or treated with dapansutrile in two different doses.

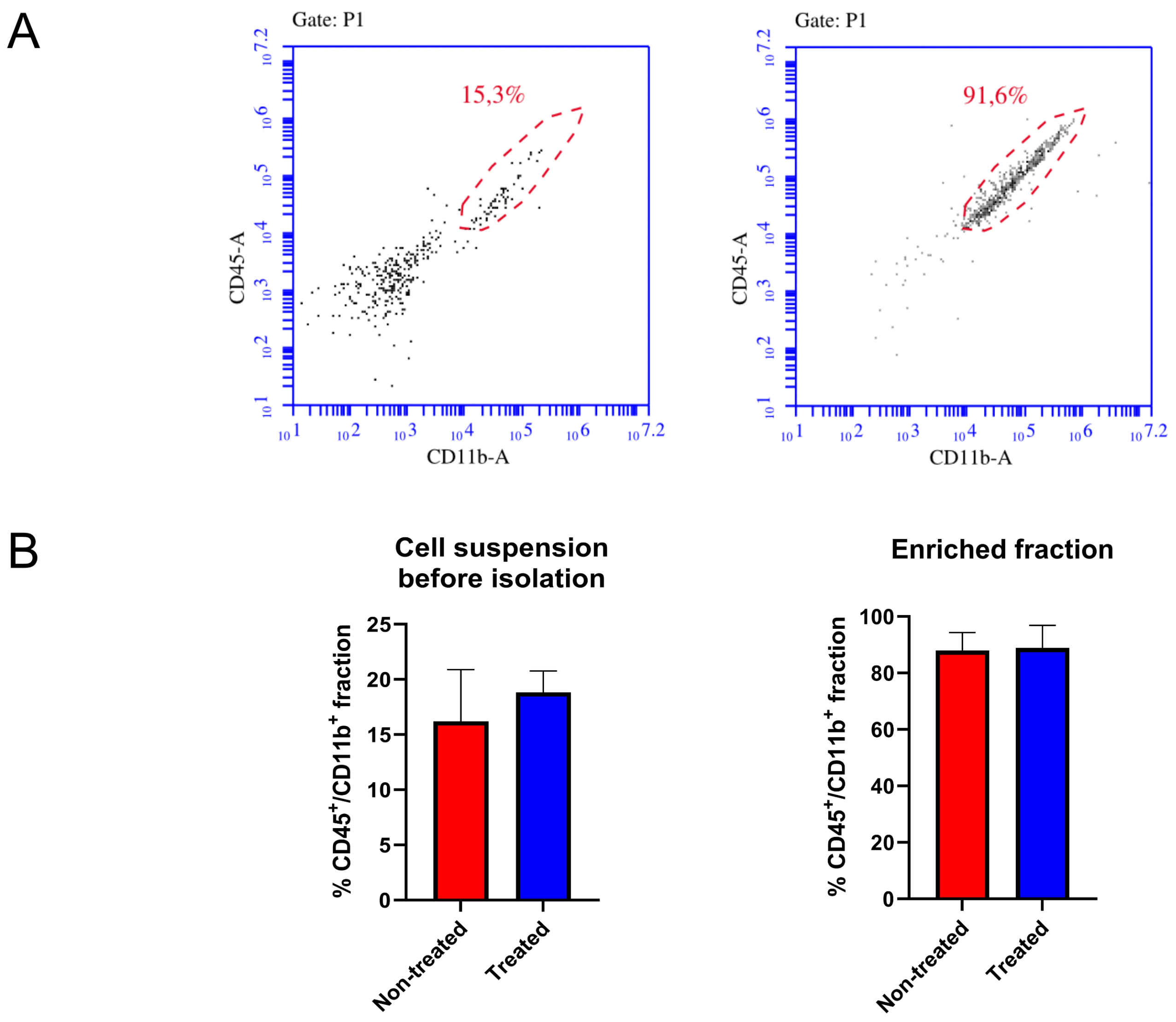

Fig. S2. Flow cytometry analysis. (A) Representative flow cytometry plots and (B) bar diagrams of percentage of CD45+/CD11b+ cells in total brain cell suspension and in the isolated enriched cell fraction used for RNAseq (n=5-6). Data shown as means ± SD.

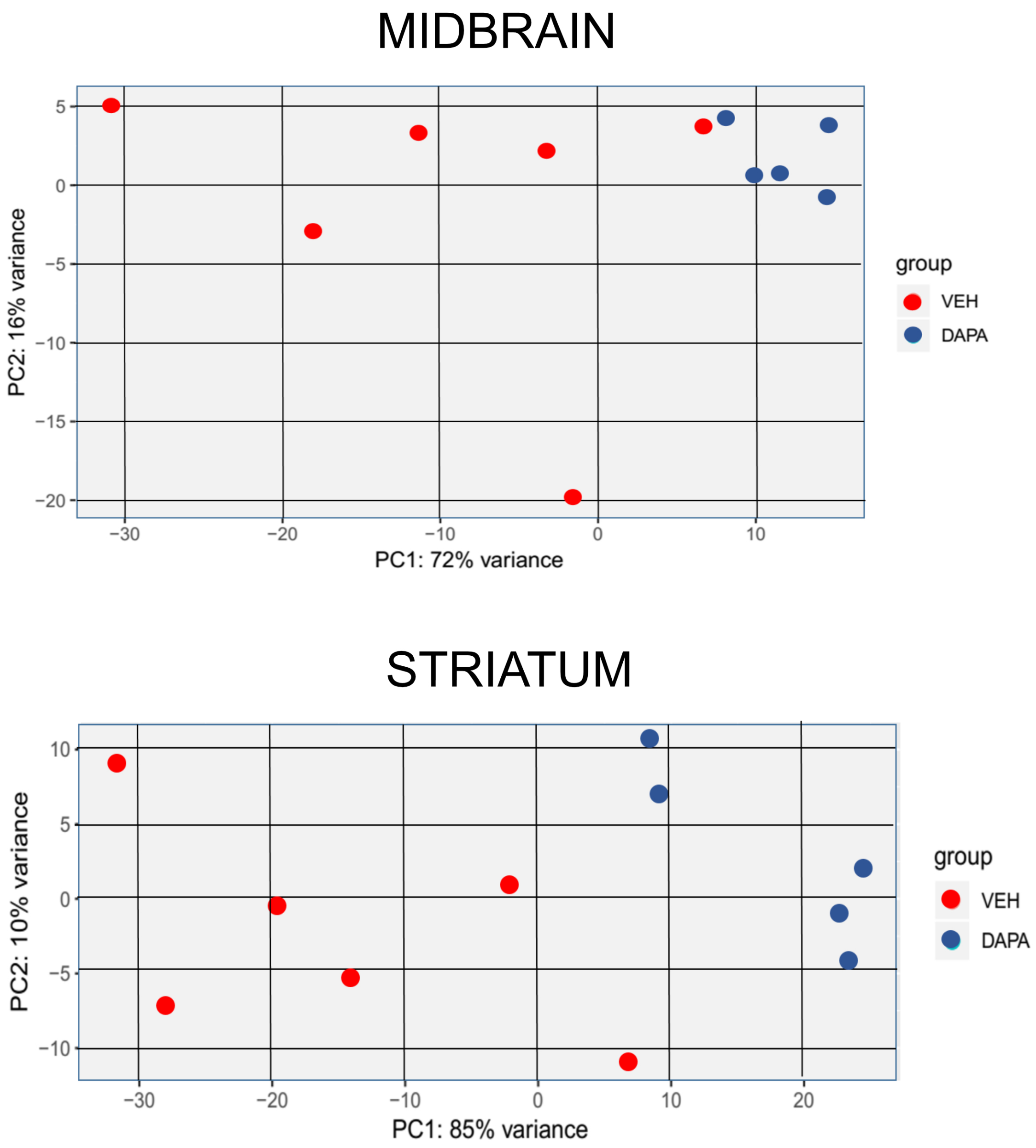

Fig. S3. Principle component analysis (PCA) plots of the raw data of RNAseq of microglia isolated from midbrain and striatum of PLP-a-syn mice, treated with vehicle (veh) or dapansutrile (dapa) as generated by DESeq2 analysis.
